# A Medical Causerie

**Published:** 1915-08-21

**Authors:** 


### A MEDICAL CAUSERIE.

One of the melancholy results of the war will be the state of mistrust and ill-feeling which will exist between members of the several belligerent nations long after the formal establishment of peace. Scientific societies and organisations will certainly not escape this harvest, as indeed is very evident from actions which have already been taken. A somewhat fervent patriotism has set itself to show that Teutonic achievements in various fields of thought and enterprise have been much over-rated, and a well-known surgeon has constituted himself a very comprehensive exponent of this doctrine. Probably a similar palace of truth has been set up in Germany in reference to our own attainments, and it is hopeless to think that the tempers which these things reveal will readily subside. Science, we have often been told, knows nothing of nationality, but on scientific men human nature evidently has a firm hold! In the natural order of things the International Medical Congress would have been held at Munich a year hence. Is it in the least likely that any such congress can be held for many years? One of the most recent examples of the penetration of scientific circles by the spirit of a fierce patriotism is the removal, by the German Laryngological Society, of the name of Sir Felix Semon from its list of honorary members. Sir Felix has long been resident in this country and has gained widespread confidence and respect. When he retired from practice some few years ago he received an impressive testimonial from his colleagues and a scholarship was founded in his honour. Everyone knows of the leading position which he occupied as a laryngological surgeon, and, as he himself has declared, he believed there was no incompatibility between loyalty to the country of his adoption and the mind and heart of a good German. The war must have put a strain on his creed, and some months back in a letter to the *Times* he expressed his detestation of the inhuman methods of German warfare. This has apparently been too much for his Teutonic brethren, and they have accordingly removed his name from their laryngological list.

The late Mr. Edmund Owen had a happy capacity for smart repartee, and many are the stories told of his power of tingling reproof as practised on the careless or ignorant student. One of the best of these tells of a student—a somewhat long stayer in the undergraduate stage—who, having answered a question correctly, was so unwise as to remark to Mr. Owen: "You seem surprised, sir." Quick came the more than sufficient response: "So was Balaam!" There was, however, at least one occasion when Owen met his match. He had in an after-dinner speech replied to the toast of "The Guests," and in mock-heroic fashion had protested against the proposer's description of him as "Dr." Owen. A later speaker, whether intentionally or unintentionally, repeated the description and then, stopping short, offered profuse apologies, and added, "I assure Mr. Owen that I fully appreciate the difference on which he insists. He will have us call him 'Mr.,' not 'Dr.'—just so, expert, not learned." Owen was a man of excellent temper, and no one enjoyed the joke more thoroughly than himself. Another medical teacher, still happily with us, is also well known for his ability to scourge his students with a ready tongue. After discussing a case at the bedside he turned to a member of the class and inquired, "Now, Mr. Jones, suppose that you had this patient under your care. What would you do for him?" Mr. Jones was evidently gravelled, and after some hesitation produced as his sole resource, "I would send him to the country." There followed the immediate comment, "I think, Mr. Jones, that if you ever have a patient you should keep him, for—you may never get another."

In these times of special difficulty Guy's Hospital and Medical School are to be congratulated on a piece of exceptional good fortune. Under the will of the late Mr. Eno the hospital, as lately recorded in our Notes, receives £25,000 for general, and £25,000 for re-endowment purposes. Evidently the combination of saline laxatives and moral sentiments must have been a highly profitable business, and some of the profits have come to a particularly useful service, an event on which all concerned may well be felicitated. From the trustees of the late Sir William Dunn, Bart., War Loan scrip to the amount of £25,000 has been transferred to the governors of the hospital for the purpose of founding a lectureship in pathology, and this, indeed, is excellent news. Guy's has possessed some great men whose fame as physicians was largely founded on pathological observation. Thus the new endowment will help to continue a great tradition in the altered circumstances of the present day. Pathology has become so much a matter of microscopy, of bacteriology, and of chemistry that no practising physician can nowadays hope to compass it. *Post-mortem* anatomy has played an important part in medicine, but further developments in this direction can hardly be expected. The claim is for laboratory work so far as research is concerned, though this is not to say that the macroscopic demonstration of diseased organs is not a highly important factor in the adequate training of the medical student and practitioner.

Indeed, as to the latter, one of the addresses given at the last International Congress on Post-Graduate Teaching urged very strongly that at all the hospitals and infirmaries throughout the land efforts should be made to give medical practitioners the opportunity of seeing *post-mortem* examinations, and the suggestion received strong support. There can be no doubt that the medical man would greatly gain had he more frequent opportunities of confirming or correcting his diagnosis by the examination of fatal cases. The prejudice which opposes this is a hindrance to the service which it is the privilege of medicine to render.

